# Alterations in the Gut Microbiome and Cecal Metabolome During *Klebsiella pneumoniae*-Induced Pneumosepsis

**DOI:** 10.3389/fimmu.2020.01331

**Published:** 2020-07-31

**Authors:** Ting Wu, Fangming Xu, Cong Su, Hongru Li, Na Lv, Yanyan Liu, Yufeng Gao, Yanhu Lan, Jiabin Li

**Affiliations:** ^1^Department of Infectious Diseases, The First Affiliated Hospital of Anhui Medical University, Hefei, China; ^2^Department of Infectious Diseases, The Chaohu Affiliated Hospital of Anhui Medical University, Hefei, China; ^3^Department of Neurology, Xiangya Hospital Central South University, Changsha, China; ^4^Department of Stomatology, The First Affiliated Hospital of Anhui Medical University, Hefei, China; ^5^Anhui Center for Surveillance of Bacterial Resistance, Hefei, China; ^6^Institute of Bacterial Resistance, Anhui Medical University, Hefei, China

**Keywords:** *K. pneumoniae* pneumonia, metabolome, gut microbiome, multi-omics analyses, short-chain fatty acids, phagocytosis

## Abstract

*Klebsiella* (*K*.) *pneumoniae* is a common cause of pneumonia-derived sepsis in human and is associated with high morbidity and mortality. The microbiota promotes and maintains host immune homeostasis during bacterial infections. However, the mechanisms by which the gut microbiota affects immune responses in the lung still remain poorly understood. Here, we performed cecal metabolomics sequencing and fecal 16s rRNA sequencing in *K. pneumoniae*-infected mice and uninfected controls and showed that *K. pneumoniae* infection led to profound alterations in the gut microbiome and thus the cecal metabolome. We observed that the levels of *Lactobacillus reuteri and Bifidobacterium pseudolongum* were significantly decreased in *K. pneumoniae*-infected mice. Spearman correlation analysis showed that alterations in the richness and composition of the gut microbiota were associated with profound changes in host metabolite concentrations. Further, short-chain fatty acids (SCFAs), including acetate, propionate, and butyrate, were detected in cecal contents and serum by gas chromatography-mass spectrometry (GC-MS). We observed that the concentrations of these three SCFAs were all lower in the infected groups than in the untreated controls. Lastly, oral supplementation with these three SCFAs reduced susceptibility to *K. pneumoniae* infections, as indicated by lower bacterial burdens in the lung and higher survival rates. Our data highlight the protective roles of gut microbiota and certain metabolites in *K. pneumoniae*-pneumonia and suggests that it is possible to intervene in this bacterial pneumonia by targeting the gut microbiota.

## Introduction

*K. pneumoniae* is a severe, multidrug-resistant (MDR) pathogen that currently has high morbidity and mortality owing to the limited availability of treatment options (1, 2). Being common natural inhabitants of our commensal microbiome, the risk of worldwide spread of these MDR pathogens has become a recognized global threat. Therefore, it is imperative to expand and deepen our understanding of how host defenses limit the dissemination and pathogenesis of these bacteria, and to develop novel therapeutic strategies.

The role of gut microbiota in mediating the host immune/inflammatory responses locally and systemically has become recognized over the last two decades. The maintenance of the host-microbiota balance is a key for homeostasis in animals. Alterations in the microbiome and metabolome and their interaction with the immune, endocrine, and mucosal systems are associated with a wide array of illnesses, and vice versa, diseases, and pathological conditions tend to cause gut microbiota dysbiosis and altered production of microbial metabolites, leading to dysregulation of the metabolism and immune system (3–7). Emerging evidence has elucidated the crucial functions of the normal microbiome in modulating various systems in the host body, and thus the concepts of the gut–brain axis (8), gut–liver axis (9), and gut–lung axis (10) have been proposed, examined, and accepted by the majority of researchers. Indeed, disruption of the gut–lung axis has already been associated with respiratory diseases (10–14), the mechanisms by which the gut microbiota affects the immune responses in distal lung, however, still remain poorly characterized.

Multiple microbiota elements, such as pattern recognition receptor ligands and diet-dependent bile acids and vitamins, have been demonstrated to maintain host immune homeostasis (8, 15–17). Considerable studies have shown that gut microbiota can induce different types of immune cells, such as Th1/17 cells and Foxp3+ regulatory T cells, and thus regulates immune responses (18, 19). In addition, the systemic effects of gut microbiota are attributed to poorly studied short-chain fatty acids (SCFAs), which are produced in the intestinal tract as end products of fiber fermentation (20) and transported to remote organs via the blood circulation. A variety of bacterial species collectively contribute to the production of acetate, propionate, and butyrate, the most abundant SCFAs *in vivo* (20). These bacterial metabolites are essential elements in the interaction between microbiota and host. Indeed, associations between changes in their production and development of inflammatory/immune disorders have been identified (21–25). However, how these metabolites are involved in sensing and modulating the immune response in remote organs is still poorly understood.

In the present study, we systematically demonstrated the protective roles of gut microbiota in the immune responses in the lung during infection by the major human pathogen *K. pneumoniae* by combining mouse models and multi-omics analyses. We analyzed cecal metabolome and gut microbiome profiles in *K. pneumoniae*-infected mice and controls and performed Spearman correlation analyses. We tried to investigate whether altered abundance of metabolites and associated gut microbiota was correlated with bacterial pneumonia. Furthermore, we investigated whether oral supplementation with SCFAs or SCFA-derived bacteria might be effective in bacterial pneumonia treatment. Our work highlights the functional effects of the gut microbiota and its associated metabolites on bacterial pneumonia.

## Materials and Methods

### Mice

All experiments involving mice were performed in accordance with the guidelines of the Institutional Animal Care and Use Committee of Anhui Medical University (No. LLSC20190253). Wild-type C57BL/6 mice were purchased from the Experimental Animal Center of Anhui Province (Hefei, China). Experimental groups were age- and sex-matched and housed in Anhui Medical University under standard care. All mice were female and between 6 and 8 weeks old.

### Bacterial Strains and Cells

*K. pneumoniae* (ATCC 43816) was cultured in Luria broth (LB) at 37°C overnight. The RAW264.7 cell line was obtained from American Type Culture Collection (ATCC) and cultured in endotoxin-free Dulbecco's modified Eagle's medium (DMEM) containing 10% fetal bovine serum (FBS, Gibco) and 1% penicillin/streptomycin (Thermo Fisher, USA).

### Reagents and Consumable Material

The main reagents and consumable materials are listed in Supplementary Table 1.

### Mouse Models of *K. pneumoniae* Infection and Antibiotic-Treatment

Mice were treated with broad-spectrum antibiotics (ampicillin, 1 g·L^−1^; neomycin sulfate, 1 g·L^−1^; metronidazole, 1 g·L^−1^; vancomycin, 0.5 g·L^−1^) dissolved in drinking water for 14 days (11, 14). Antibiotic treatment was stopped 3 days prior to infection. Then, mice with or without antibiotic treatment were anesthetized with isoflurane and inoculated intranasally with 1 × 10^5^ CFU of *K. Pneumoniae* in 50 μL of phosphate-buffered saline (PBS) and were sacrificed at different time points after infection.

### Fecal Microbiota Transplantation (FMT)

Fecal samples from randomly chosen healthy mice were used to colonize the guts of antibiotic-treated mice. Briefly, the antibiotic-treated mice and ‘healthy microbiota’ recipient mice were separately bred in different gnotobiotic isolators for 1–2 weeks. Then, mice were anesthetized with isoflurane and inoculated intranasally with 1 × 10^5^ CFU of *K. pneumoniae* in 50 μL PBS.

### SCFAs Measurement Assay

Concentrations of SCFAs in cecal contents and serum were measured by GC-MS as described previously (26). Briefly, 800-μL sample aliquots were filtered, and the protein-free filtrate was stored at −20°C before vacuum distillation. The distillation was performed using a 225-μL protein-free sample with 25 μL of internal standard solution added. Next, gas chromatography was performed, and SCFA concentrations were determined.

### SCFA Treatment Assays

As described previously (27, 28), groups of 8 or 10 wild-type mice were pre-treated with SCFAs (acetate, propionate, and butyrate) (Sigma-Aldrich) for 7 days at a concentration of 150 mM administered in drinking water (fresh solutions three or four times a week). Then, mice were anesthetized with isoflurane and inoculated intranasally with 1 × 10^5^ CFU of *K. pneumoniae* in 50 μL PBS. SCFAs (acetate, propionate, and butyrate) were added to RAW264.7 cells *in vitro* for 24 h at concentrations of 1, 10, 20, 50, and 100 times in peripheral blood of healthy people, as described previously (29, 30). Then, RAW264.7 cells were infected with *K. pneumoniae* at a MOI (multiplicity of infection) of 1:100.

### Isolation of Alveolar Macrophages

Alveolar macrophages were isolated as described previously (31). Briefly, mice were sacrificed and immediately exsanguinated. Bronchoalveolar lavage fluid (BALF) was collected with 4 ml of 37°C Dulbecco's phosphate-buffered saline (DPBS) with 0.5 mM EDTA through a 1.7-mm catheter in a 1-mL syringe. Cells were pelleted and resuspended in RPMI supplemented with 5.0% (vol/vol) fetal bovine serum, 100 U·mL^−1^ penicillin, and 100 U·mL^−1^ streptomycin, and alveolar macrophages were allowed to adhere to a tissue culture flask for 2 h (37°C, 5% CO_2_, vol/vol). In general, the alveolar macrophage purity was more than 90%, as analyzed by subsequent flow cytometry (FACS Celesta, BD Biosciences).

### Phagocytosis Assays *in vitro*

To begin with, a suspension of bacteria was suspended in PBS (pH = 9.0) and marked with CFSE (Invitrogen, Breda, the Netherlands) while stirring at 37°C for 30 min or room temperature for 2 h. Meanwhile, RAW264.7 cells or alveolar macrophages were incubated in complete medium without antibiotics for at least 5 h and then infected with *K. pneumoniae* at a MOI of 100. After 1 h of incubation, cells were subsequently washed with cold complete medium without antibiotics but supplemented with 0.05% gentamicin (50 mg/mL) and resuspended in FACS-buffer, and they were then analyzed by flow cytometry (FACS Celesta, BD Biosciences). The phagocytosis index of each sample (30,000 events) was calculated as mean fluorescence intensity (MFI) × percentage (%) positive cells (37°C)—(MFI × % positive cells; 4°C).

### DNA Extraction and 16S rRNA Sequencing

Fecal samples were collected from the mice before sacrifice. These were frozen immediately following collection and stored at −80°C prior to analysis. The fecal samples were pulverized with a mortar and pestle in liquid nitrogen, and bacterial genomic DNA was then extracted with the QIAamp DNA Stool Mini Kit (Qiagen). V4 region amplicon sequencing (515F-GTGCCAGCMGCCGCGGTAA and 806R-GGACTACHVGGGTWTCTAAT) (32) of the 16S rRNA gene was performed on an Illumina HiSeq 2500 at Beijing Genomics Institute (BGI-Shenzhen, China). The mothur database (http://www.mothur.org/) (33) was used to obtain unique reads. Sequences of <200 and >1,000 bp as well as sequences containing any primer mismatches, barcode mismatches, ambiguous bases, and homopolymer runs exceeding six bases were all excluded. All remaining sequences were assigned to operational taxonomic units (OTUs) with a 97% threshold of pairwise identity and then classified taxonomically using the RDP database (http://www.mothur.org/wiki/RDP_reference_files) (34). These taxonomies were then used to construct summaries of the taxonomic distributions of OTUs, which can be applied to calculate the relative abundances of microbiota at different levels.

### Metabolome Sequencing of Cecal Contents

Cecum samples were subsequently extracted on mouse death and then frozen immediately at −80°C. For metabolomics profiling, all cecal content samples were thawed on ice and a quality control (QC) sample, made by mixing and blending equal volumes (10 μL) of each cecal content sample, was used to estimate a mean profile representing all the analytes encountered during analysis. We isolated and extracted metabolites (<1,500 Da) as follows. Firstly, 100-μL cecum mixtures were precipitated with 200 μL methanol, and similarly, the QC sample was precipitated with methanol (1:2 v/v). All samples were subsequently centrifuged at 14,000 × g for 10 min at 4°C. The supernatants were subjected to metabolomics profiling by liquid chromatograph mass spectrometer (LC-MS) at the Beijing Genomics Institute (BGI-Shenzhen, China). Pretreatments of the acquired MS data, including peak selection and grouping, retention time correction, second peak grouping, and isotope and adduct annotation, were performed as previously described (35). LC-MS raw data files were converted into mzXML format and then analyzed by the XCMS and CAMERA toolbox with the R statistical language (v3.4.1). The online KEGG database (http://www.genome.jp/kegg/) (36) and the HMDB database (http://www.hmdb.ca) (37) were used to identify different metabolites. If the mass difference between the observed and theoretical mass was <10 ppm, the metabolite name was reported, and the molecular formulas of the matched metabolites were further validated by isotopic distribution measurements. Commercial reference standards were used to validate and confirm metabolites by comparison of their retention times and MS/MS spectra.

### RNA Extraction, Reverse Transcription, and Quantitative Real-Time PCR

RNA was extracted from RAW264.7 cells or alveolar macrophage homogenates using the RNeasy Plus Mini Kit (Qiagen, cat. no. 74134) by strictly following the manufacturer's protocol. For reverse transcription, single-strand cDNA was synthesized using PrimeScript first strand cDNA Synthesis Kit (TaKaRa, cat. no. D6110A). Real-time PCR was performed using PrimeScript^TM^ RT Master Mix (Takara, cat. no. RR036A) in a Three-Step Real Time PCR System (Light Cycler 96). The target gene expression levels were normalized to the housekeeping gene (*Gapdh*) mRNA according to the 2^−ΔΔCt^ calculation method. The primers for real-time PCR were as follows: *Tnf*α-forward: 5′-ACTGAACTTCGGGGTGATCG-3′, *Tnf*α-reverse: 5′-TTGAGATCCATGCCGTTGGC-3′; *1L-1*β-forward: 5′-CATCCAGCTTCAAATCTCGCA-3′, *1L-1*β-reverse: 5′-GATGAAGGAAAAGAAGGTGCTC-3′; *1L-6*-forward: 5′-ACTTCACAAGTCGGAGGCTTA-3′, *1L-6*-reverse: 5′-ATCCAGTTTGGTAGCATCCATC-3′; *Mcp-1*-forward: 5′-AGTAGGCTGGA GAGCTACAAGA-3′, *Mcp-1*-reverse: 5′-TGCTGAAGACCTTAGGGCAGAT-3′; *Cxcl1*-forward: 5′-ACAGGGGCGCCTATCGC-3′, *Cxcl1*-reverse: 5′-ACAAT TTTCTGAACCAAGGGAGC′; *Gapdh*-forward: 5′-GTCAAGGCCGAGAATGGGAA-3′, *Gapdh*-reverse: 5′-CTCGTGGTTCAC ACCCATCA-3′.

### Flow Cytometry

For RAW246.7 cells or alveolar macrophages, flow cytometry was conducted as follows. Briefly, 500 μL of cold 50 mM ethylenediaminetetraacetic acid (EDTA) in PBS was added in each well and incubated for at least 30 min at 37°C in 5% CO_2_ in a cell culture incubator. Subsequently, the cells were transferred to FACS tubes and centrifuged at 1,000 × g for 10 min. The supernatant was carefully removed, and the cell pellet was resuspended in 200 μL of freshly prepared staining solution. The samples were incubated in the dark for 25 min, and subsequently, 200 μL of FACS buffer (2 mM EDTA in 10% PBS) was added, followed by analysis with a FACS Calibur flow cytometer (FACS Celesta, BD Biosciences). Electronic compensation was used to eliminate bleed-through fluorescence.

### Statistical Analysis

All results are presented as the mean ± SEM. Statistical analysis was performed using unpaired, Student's *t*-tests for two groups and one-way analysis of variance (ANOVA) or two-way ANOVA for multiple groups. Sample sizes were selected on the basis of preliminary results to ensure an adequate power. The study and experiments were not randomized or blinded. Results were considered statistically significant or extremely significant when *P*-values were <0.05 or 0.01, respectively. All graphs were generated using the Adobe Illustrator CC 2017 release or Graph Pad Prism 7.

## Results

### Alterations in the Cecal Metabolome of *K. pneumoniae*-Infected Mice

**Figure 1:**
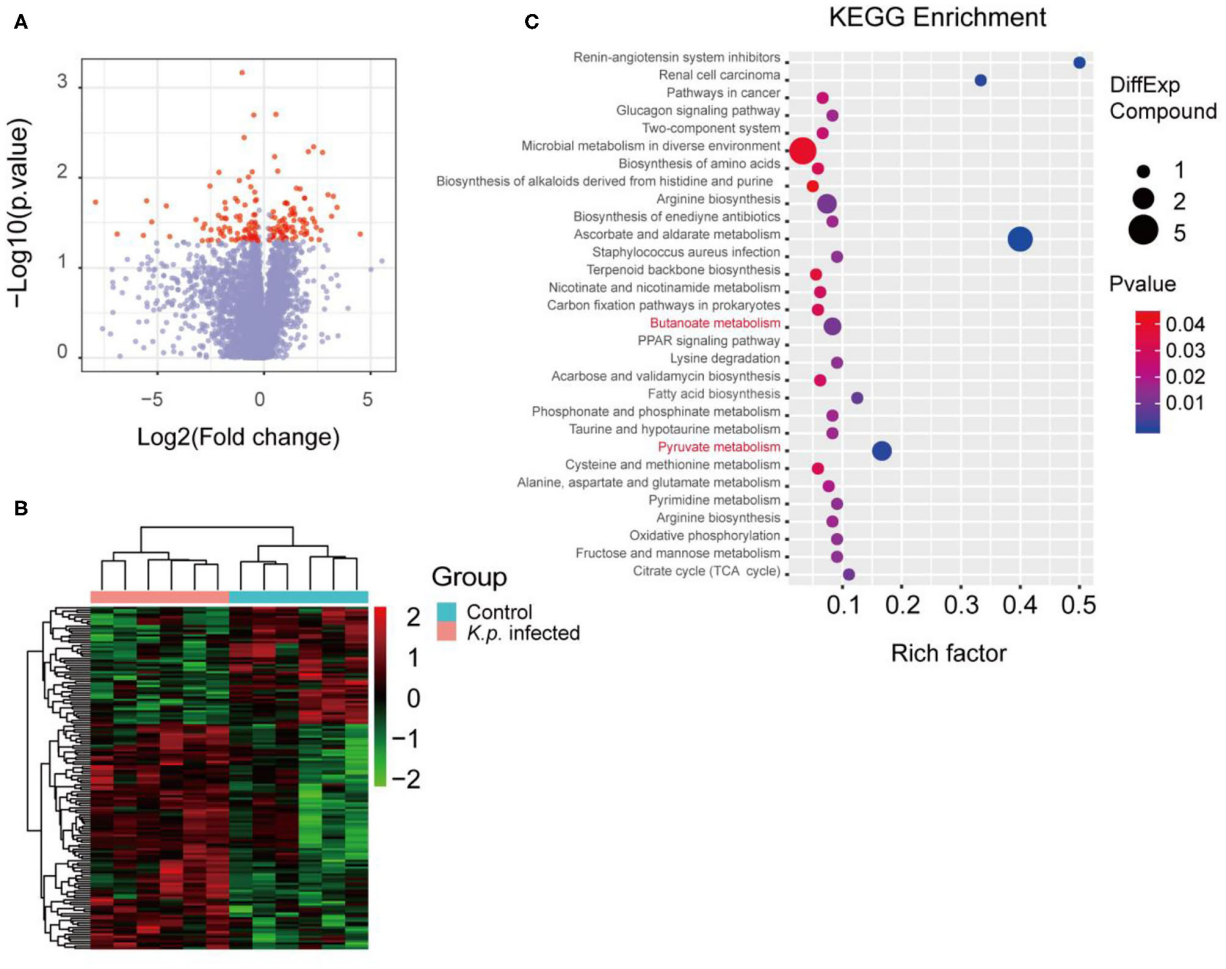
Metabolomic profiles of cecal contents in *K. pneumoniae*-infected mice and healthy controls. **(A)** Volcano plot for analyses of host-produced metabolites in cecal contents from *K. pneumoniae-*infected mice and untreated controls. Fold change ≥ 1.5 or ≤0.5 and *p* < 0.05 are marked in red, and the rest are in blue. **(B)** Hierarchical clustering heatmap of host-produced metabolites in cecal contents of *K. pneumoniae-*infected mice and untreated controls. Red denotes increased expression, while green denotes decreased expression. **(C)** Selected examples of KEGG pathway enrichment. Disturbances in SCFA metabolism are highlighted in red. Group size is 8–10 per group. *P-*values are determined using two-tailed Student's *t*-tests **(A)** or the Hypergeometric test and Benjamini–Hochberg FDR correction **(C)**.

The mammalian immune system has a complex and dynamic bidirectional relationship with the microbiome (38). As previously described, the gut microbiota regulates the host immune systems, in part by producing metabolites (39, 40). To assess the specific metabolomic profiles of *K. pneumonia-*challenged mice, we performed in-depth untargeted metabolomics sequencing of cecal contents by liquid chromatography–mass spectrometry (LC-MS) at Beijing Genomics Institute, Shenzhen. The sample preparation and detection conditions were optimized to account for the variable stability of metabolites and ensure that all possible metabolites were collected from the cecal contents. To sum up, we identified, based on the Kyoto Encyclopedia of Genes and Genomes (KEGG) database, 149 metabolites that differed in abundance between *K. pneumoniae-*infected mice and uninfected controls, including both host-derived and bacterially derived metabolites (Figures 1A,B). These cecum metabolites, which were primarily associated with carbohydrate and amino acid metabolism, were then grouped into 72 co-abundance clusters across all subjects, and selected examples were listed (Figure 1C). Intriguingly, we observed that the abundance of SCFAs (short-chain fatty acids) was significantly decreased, which we speculated would attenuate the host antimicrobial activity. Additionally, we also analyzed the metabolomics profiles between *K. pneumoniae-*infected, antibiotic-treated mice and antibiotic-treated controls to further explore the roles that gut microbiota played (Supplementary Figures 1A,B). Similar to the results above, we also observed that signal pathways related to SCFA metabolism were dysregulated. Taken together, we propose that this indicates that *K. pneumoniae* infection, whether the mice are antibiotic-treatment or not, will change metabolic profiles compared with healthy controls.

### Alterations in the Gut Microbiome of *K. pneumoniae*-Infected Mice

**Figure 2:**
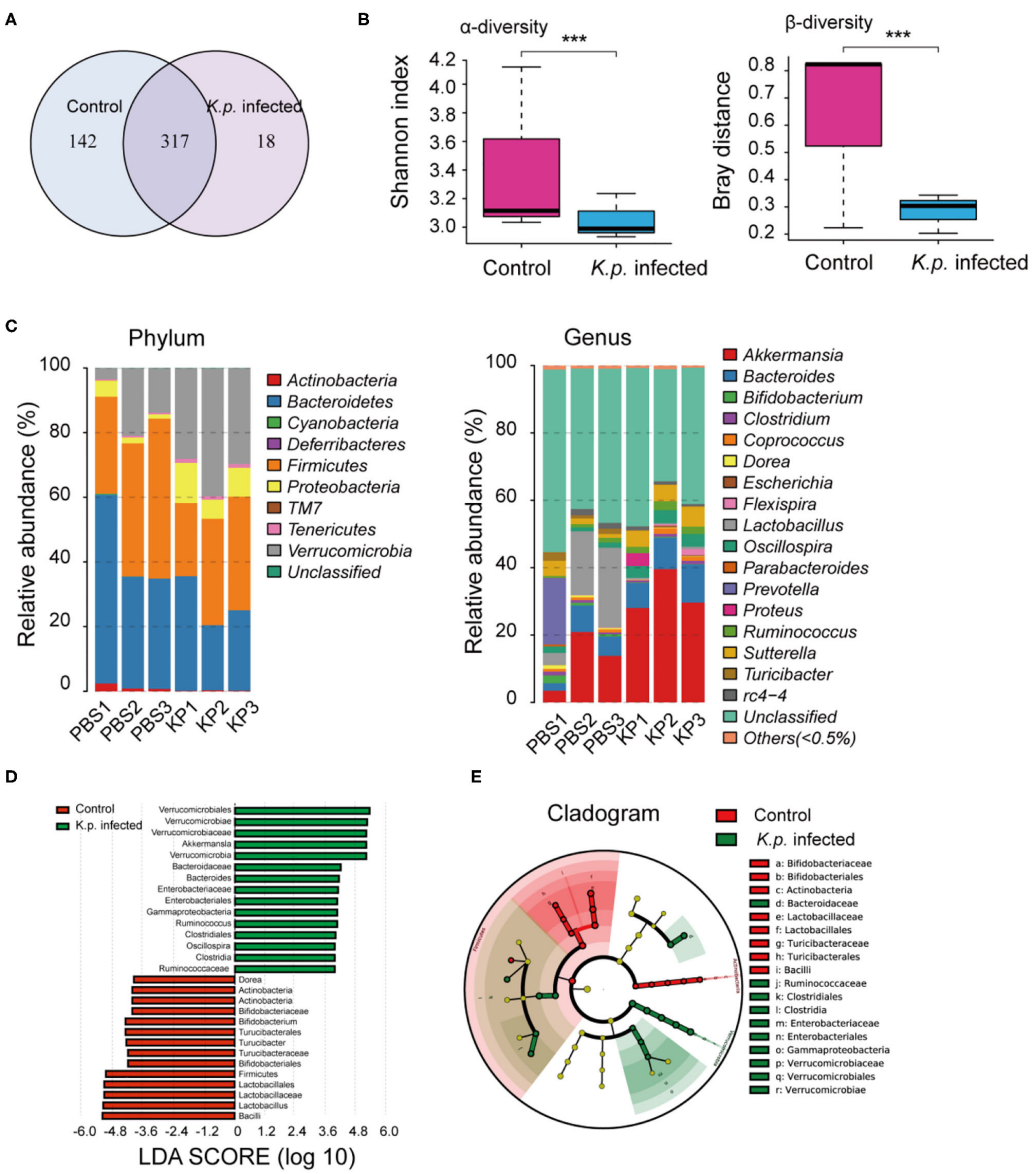
Comparison of gut microbiome between *K. pneumoniae*-infected mice and healthy controls. **(A)** Venn diagram of operational taxonomic units (OTUs) in *K. pneumoniae-*infected mice and untreated controls. **(B)** α-diversity (Shannon index) and β-diversity (Bray–Curtis similarity index) of 16S rRNA genes from *K. pneumoniae-*infected mice and untreated controls. **(C)** Discriminative OTUs abundance of taxonomic distributions at phylum and genus levels in *K. pneumoniae-*infected mice compared to untreated controls. **(D)** LEfSe analysis shows differentially abundant genera as biomarkers between *K. pneumoniae-*infected mice and untreated controls. **(E)** Cladogram representation of the differentially abundant families and genera between *K. pneumoniae-*infected mice and untreated controls. Group size is 8–10 per group. *P*-values are determined by two-tailed Wilcoxon rank-sum test **(B)** and Kruskal–Wallis test **(D)** (*P* < 0.05). ***P < 0.001.

To identify specific microbiota species or genera associated with *K. pneumoniae* pneumonia and determine whether the altered abundance of circulating metabolites was correlated with specific gut microbiota, we performed 16S ribosomal RNA sequencing on the basis of V4 variable regions. We first compared microbial differences between mice with bacterial pneumonia and controls. We obtained a total of 736,749 high-quality 16S rRNA gene sequences, which were then clustered into operational taxonomic units (OTUs) at a 97% similarity level (Figure 2A). Phylogenetic diversity analysis showed remarkable differences in the richness and diversity of *K. pneumoniae-*infected mice compared to untreated controls (Figure 2B), and the composition of bacterial communities also changed with a significant difference (Figure 2C). At the phylum level, Bacteroidetes, Firmicutes, and Verrucomicrobia dominated the fecal microbial communities of both groups. Compared with healthy controls, mice infected with *K. pneumoniae* pneumonia had fewer Bacteroidetes and Firmicutes but higher levels of Proteobacteria and Verrucomicrobia. At the genus level, *Bacteroides* was the dominant phylotype in both groups but was significantly decreased in the *K. pneumoniae*-infected groups compared with the uninfected controls. Notably, *Parabacteroides, Bifidobacterium, Clostridium, Coprococcus*, and *Prevotella*, at the genus level, which produce SCFAs, were all decreased in the *K. pneumoniae-*infected mice. Next, we examined whether specific gut microbiota species were associated with *K. pneumoniae* infections. We observed that the levels of *Lactobacillus reuteri* and *Bifidobacterium pseudolongum*, which promote SCFA production *in vivo*, were both decreased, while those of *Escherichia coli* and *Clostridium methylpentosum* were increased in *K. pneumoniae-*infected mice compared with uninfected mice (Table 1). We performed a linear discriminant analysis effect size (LEfSe) analysis to identify specific taxa that varied in abundance and that would potentially be used as biomarkers. In total, 29 genera were identified with LDA scores >3.5 (Figure 2D). A cladogram for family- and genus-level abundance is shown in Figure 2E. Moreover, similar alterations in the gut microbiome were observed when all mice were treated with antibiotics (Supplementary Figures 2A–C), and *K. pneumoniae* infection had a deeper impact on the gut microbiome. To explore the impact the gut microbiota has on *K. pneumoniae* infection further, we performed fecal microbiota transplantation (FMT) into antibiotic-treated mice. We observed that pulmonary pathological scores were improved in antibiotic-treated mice on the 2nd week post-FMT (Supplementary Figure 2D). On the basis of these data, we concluded that mice with *K. pneumoniae*-induced sepsis were associated with disruption of the gut microbiome whether treated with antibiotics or not.

**Table 1:** Discriminative OTU abundance of taxonomic distributions at species levels in *K. pneumoniae*-infected mice compared to untreated controls.

**Species**	**Mean ± SD** **(Control)**	**Mean ± SD** **(*K. p*. infected)**	**Fold change**	***P*-value**
*Lactobacillus_reuteri*	1.35 ± 1.62	0.05 ± 0.04	27	0.01
*Bifidobacterium_pseudolongum*	1.27 ± 0.90	0.19 ± 0.06	6.6	0.01
*Butyricicoccus_pullicaecorum*	0.05 ± 0.05	0.02 ± 0.00	2.5	0.01
*Akkermansia_muciniphila*	12.73 ± 8.77	32.42 ± 6.24	2.55	0.01
*Clostridium_cocleatum*	0.16 ± 0.13	0.38 ± 0.08	2.38	0.01
*Clostridium_methylpentosum*	0.034 ± 0.01	0.37 ± 0.07	10.9	0.01
*Bacteroides_fragilis*	4.94 ± 3.43	9.35 ± 1.95	2	0.02
*Escherichia_coli*	0.01 ± 0.03	0.26 ± 0.25	26	0.03
*Ruminococcus_gnavus*	0.52 ± 0.35	0.90 ± 0.41	2	0.04

### Correlations Between the Cecal Metabolome and Gut Microbiome

**Figure 3:**
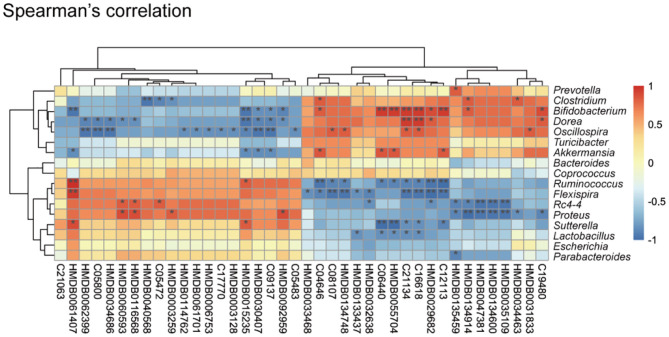
Correlation between the cecal metabolome and fecal gut microbiota. Spearman's rank correlation between cecum metabolites and associated gut microbiota in *K. pneumoniae-*infected mice and untreated controls. Red connections indicate a positive correlation, while blue connections are negative. *P-*values are determined by two-tailed Wilcoxon rank-sum test. *P < 0.05, **P < 0.01.

An altered cecum metabolic profile reflecting differences in the gut microbiome of controls and respiratory infection has recently been demonstrated (41). To further investigate the relationships between gut microbiota and cecal metabolites, we performed a Spearman correlation analysis between 149 metabolites and nine genus-level bacterial taxa (Figure 3). We identified 34 statistically significant interactions between 40 metabolites and nine bacterial taxa. This suggested that the observed alterations in cecal metabolites upon *K. pneumoniae* infection were likely derived from the gut microbiota. Taken together, the observed strong correlations between the gut microbial changes and shifted metabolic levels indicated that this bacterial pneumonia can result in significant changes in the gut microbiota, which result in dramatic shifts in host metabolite abundance, thus leading to dysregulation of host immune response to pulmonary bacterial infection.

### SCFA Measurement in *K. pneumoniae*-Infected Mice by GC-MS

**Figure 4:**
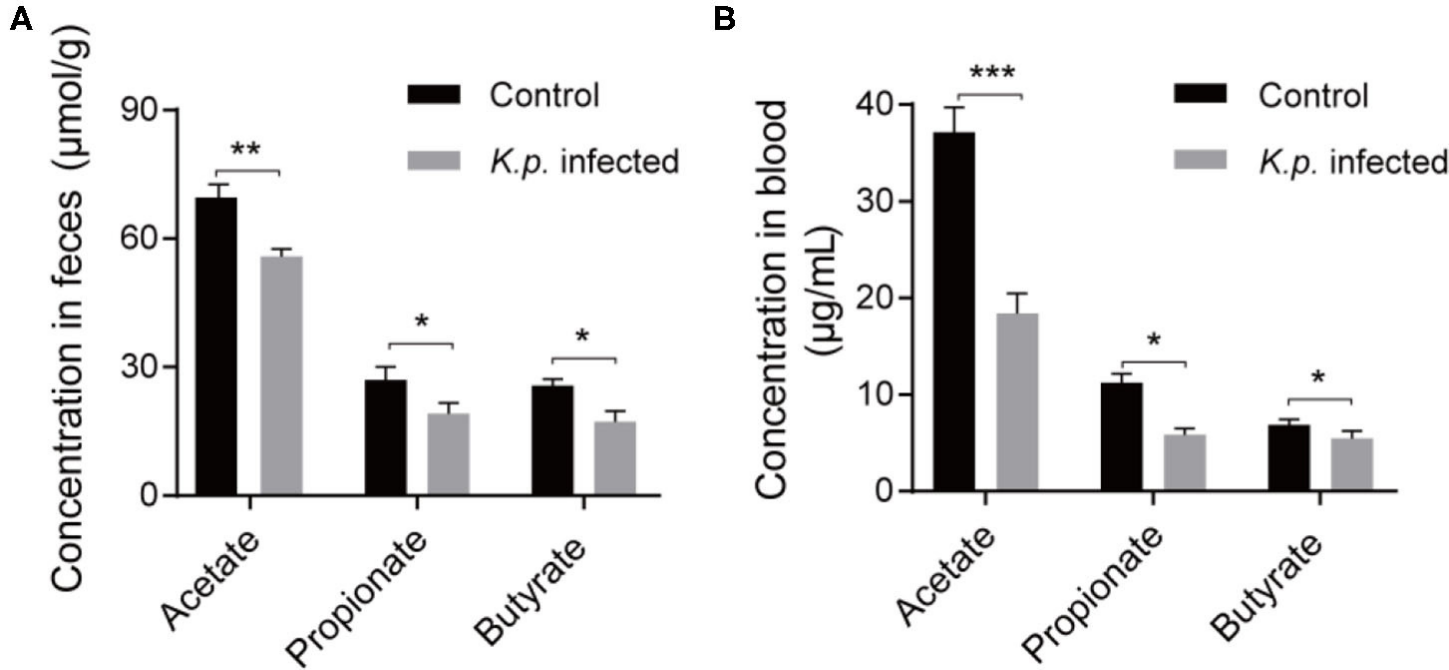
Detection of absolute concentration of SCFAs in *Klebsiella* pneumoniae mice and healthy controls. **(A)** The absolute concentration of SCFAs in cecal contents from *K. pneumoniae*-infected mice and controls. **(B)** The absolute concentration of SCFAs in serum from *K. pneumoniae*-infected mice and controls. Data are from one experiment representative of three independent experiments (mean ± s.e.m). Group size is 8–12 per group. *P-*values are determined using two-tailed Student's *t*-tests **(A,B)**. *P < 0.05, **P < 0.01, ***P < 0.001.

Recent studies have proposed that the vital link between microbiota and host immune response is due to the production of SCFAs through bacterial metabolism (42, 43). These molecules can be transported to remote organs via circulation. To further investigate the concentrations of SCFAs in cecal contents and serum, we took randomly selected samples from the infected groups and controls, extracted SCFAs, and performed detection by using a gas chromatography–mass spectrometry (GC-MS)-based metabolomics approach. We detected the three most abundant SCFAs (Figures 4A,B): acetate, propionate, and butyrate. As expected, the concentrations of all three of these SCFAs were higher in the control groups than in the infected groups. Acetic acid was the most abundant, followed by propionate, and butyrate was the least abundant. These data demonstrate that this bacterial pneumonia causes decreases in SCFAs.

### SCFAs Ameliorate *K. pneumoniae*-Induced Pulmonary Inflammation

**Figure 5:**
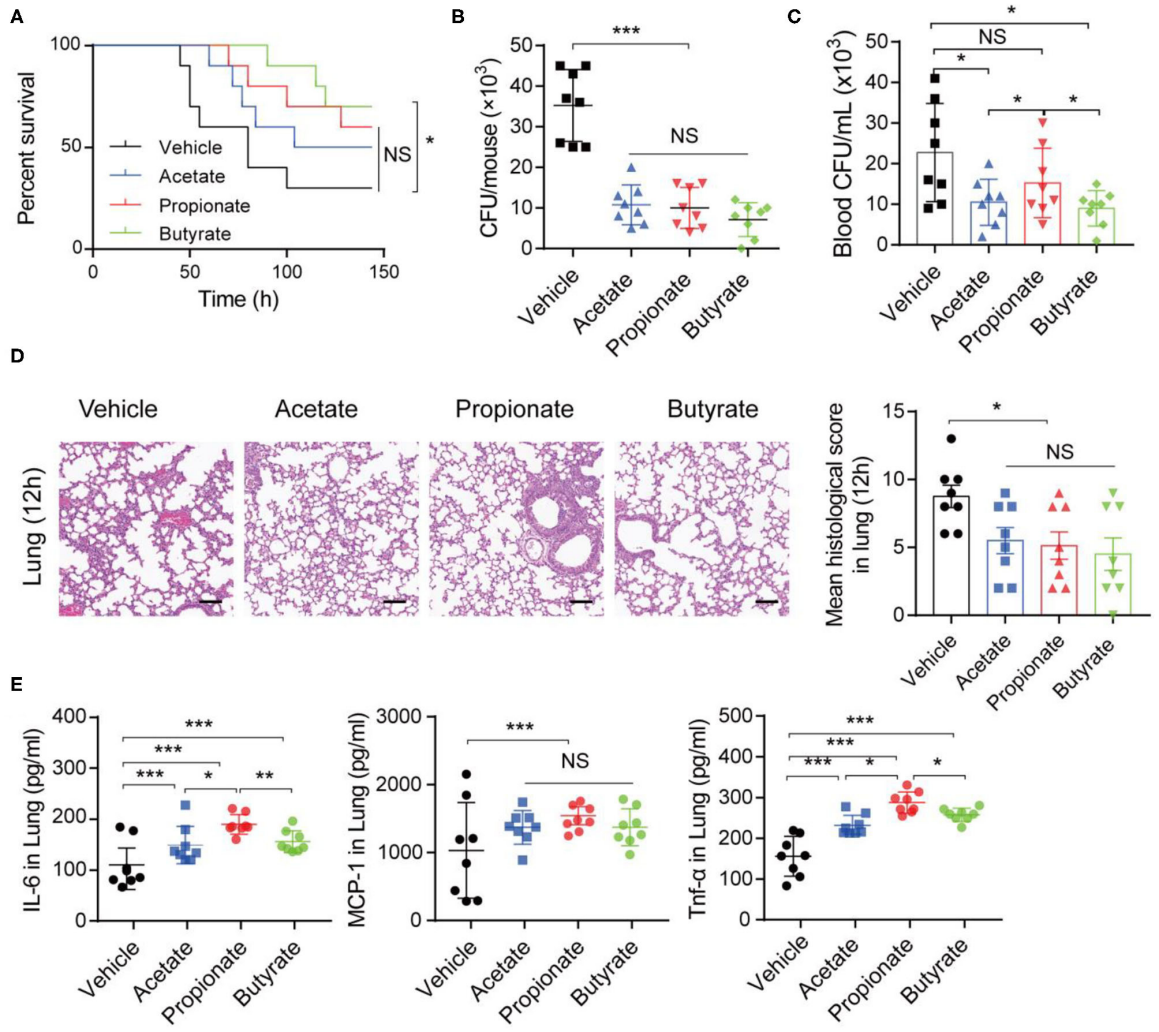
SCFAs promote inflammatory responses during *K. pneumoniae*-induced sepsis. **(A)** Survival rates of SCFA-treated mice and untreated controls post 1 × 10^5^ CFU of *K. pneumoniae* infection. **(B)** Lung and blood **(C)** bacterial burdens in SCFA-treated mice and untreated controls 12 h post *K. pneumoniae* infection. **(D)** Representative H&E staining and quantification of pathological scores of lung sections from SCFA-treated mice and untreated controls 12 h post *K. pneumoniae* infection. Scale bars: 50 μm. **(E)** Detection of cytokine production in the lung of mice 24 h post *K. pneumoniae* infection by CBA. Data are from one experiment representative of three independent experiments (mean ± s.e.m). Group size is 8–12 per group. *P*-values are determined by two-tailed Student's *t*-tests **(B–E)** or Log-rank (Mantel–Cox) test **(A)**. *P < 0.05, **P < 0.01, ***P < 0.001. NS, not significant.

To further assess the impact of SCFAs on *K. pneumoniae-*induced pneumonia, we performed *in vivo* and *in vitro* experiments. Three SCFAs (acetate, propionate, and butyrate) were administered in drinking water for 7 days before *K. pneumoniae*-challenge. Notably, bacterial infection-induced survival rates were lower in SCFA-treated mice (Figure 5A). Furthermore, supplementation with exogenous SCFAs significantly reduced the bacterial burdens in the lungs (Figure 5B) and blood (Figure 5C). In addition, SCFA-treated mice showed improved pulmonary histological pathology 12 h post *K. pneumoniae* infection (Figure 5D). As expected, oral supplementation with SCFAs increased the production of inflammatory cytokines, such as interleukin (IL)-6 and tumor necrosis factor (TNF)-α, and chemokines, such as monocyte chemoattractant protein (MCP)-1, in the lung 24 h after *K. pneumoniae* infection (Figure 5E). These data demonstrated that SCFAs help control the inflammation induced by *K. pneumoniae* infection.

### SCFAs Enhance Macrophage Function

**Figure 6:**
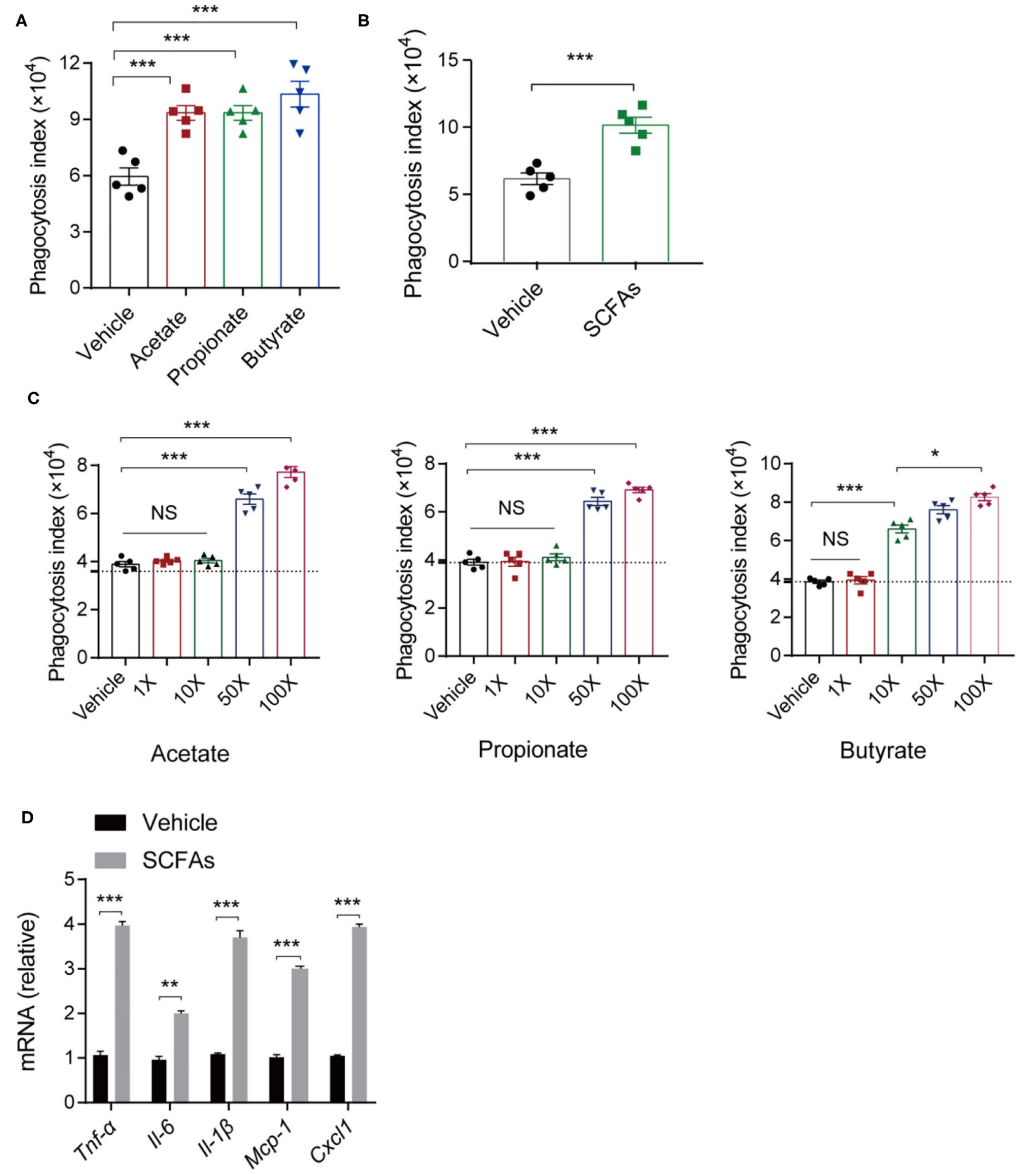
*In vitro* and *in vivo* analyses of macrophage phagocytosis of *K. pneumoniae*. **(A)** Phagocytic capacity of different SCFA-treated mice and untreated controls post *K. pneumoniae* infection. **(B)** Phagocytic capacity of isolated alveolar macrophages from 150-mM SCFA-treated mice and untreated controls. **(C)** Phagocytic capacity of different concentrations of SCFA-treated RAW264.7 and untreated controls (1× acetate: 200 μm, 1× propionate: 5 μm, 1× butyrate: 5 μm). **(D)** RT-qPCR analysis of *Tnf-*α, *Il-6, Il-1*β, *Mcp-1*, and *Cxcl1* mRNA expression in SCFA mixture-treated RAW264.7 cells and untreated controls 6 h post *K. pneumoniae* infection (MOI = 10). Group size is 8–12 per group. Data are from three independent experiments (mean ± s.e.m) **(A–D)**. *P-*values are determined using two-tailed Student's *t*-tests **(B,D)** or one-way ANOVA for multiple comparisons **(A,C)**. *P < 0.05, **P < 0.01, ***P < 0.001. NS, not significant.

The innate immune system acts as the first line of defense against invading microorganisms, and macrophages residing in lung are the key regulators of host innate immunity during bacterial infection and pneumonia (14, 44). To assess the impact of SCFAs, we analyzed the effects of different concentrations of acetate, propionate, and butyrate individually on macrophage phagocytosis. We treated C57BL/6 mice with a daily oral supplementation of 150 mM of one or more of the SCFAs for 7 days. Then, alveolar macrophages were harvested and subjected to bacterial phagocytosis assays. Alveolar macrophages from mice treated with single SCFAs or with SCFA mixtures exhibited greater phagocytosis indices for *K. pneumoniae* compared with macrophages from control mice (Figures 6A,B). There were no significant differences in the effects of the three SCFAs (acetate, propionate, and butyrate) on phagocytosis.

To test whether the effects of SCFAs on macrophage phagocytosis are relevant *in vitro*, we stimulate RAW 264.7 cells with SCFAs (acetate, propionate, and butyrate) at concentrations that were 1, 10, 50, and 100 times the concentration of SCFAs in the peripheral blood of healthy individuals. We found that macrophage phagocytosis *in vitro* was related to the SCFA concentrations. After 24 h of incubation, lower concentrations (1, 10 times higher) of SCFAs exerted little influence on the macrophage phagocytosis of *K. pneumoniae*, while higher SCFA concentrations (50, 100 times higher) enhanced macrophage phagocytosis (Figure 6C). Consistent with the *in vivo* results, 1L-1β, IL-6, TNF-α, CXCL1, and MCP-1 were all expressed at higher levels in RAW264.7 cells treated with SCFAs, as determined by quantitative real-time polymerase chain reaction (qRT-PCR) (Figure 6D). Taken together, these data suggested that SCFAs enhanced host macrophage responses to *K. pneumoniae* infection.

## Discussion

Pneumonia still remains a leading cause of death and hospitalization worldwide, especially among children and the elderly (1, 45). *K. pneumoniae* is one of the most frequently isolated pathogens in pneumonia resulting in sepsis (46, 47). As previously demonstrated, early, and aggressive antibiotic treatments exerted significant control on this bacterial infection (48, 49) or will lead to the development of MDR or XDR strains. Recent breakthroughs in our understanding of gut microbiota have important implications for respiratory disease. In the present study, we showed that alterations in the richness and composition of gut microbiota in response to infection with *K. pneumoniae* resulted in profound changes in the host metabolite abundances and observed that *Lactobacillus reuteri and Bifidobacterium pseudolongum*, at the species level, were significantly decreased in *K. pneumoniae*-infected mice, which could be further identified as potential biomarkers for this bacterial pneumonia. The levels of three SCFAs, acetate, propionate, and butyrate, were decreased in mice feces and blood samples. Acetic acid was present at the highest concentrations, followed by butyrate, and propionic acid was the least abundant. Oral supplementation with these three gut microbiota-derived metabolites SCFAs reduced susceptibility to *K. pneumoniae* infections, as demonstrated by decreased bacterial burdens in lung and higher survival rates.

It has been now becoming apparent that the effects of the large communities of intestinal microbes are not restricted to fine-tuning local innate cell function in the mucosa but also exert a systemic influence on effector cells of the innate immune system located in extraintestinal organs or tissues (50–52). The mechanistic basis for these distal influences, however, has been incompletely characterized. Previous studies have shown increased mortality from bacterial infection in the lung in the absence of the microbiota (53, 54), although specific immune defects related to this outcome are poorly understood. In the lung, several studies have shown that resistance to multiple bacterial and viral pathogens is enhanced by the microbiota (13, 14). Thus, it is of importance of exploring the mechanistic basis of how intestinal microbes reprogram lung defenses to improve the immune responses to invasion by extracellular pathogen invasion. Trompette et al. (10) first established the existence of a gut–lung axis in allergic airway disease by showing that metabolism of dietary fibers by the commensal microbiota influences the severity of allergic inflammation. Then, Clarke et al. (55) took a big step forward by demonstrating that microbiota depletion caused significant defects in the early innate response to lung infection by the major human pathogen *K. pneumoniae* and proposed that NOD-like receptor ligands were required to facilitate early bacterial clearance from the lung. In addition, some elegant studies noted that alveolar macrophages are key watchmen that constantly patrol and monitor lung tissue (56). Here, we carried out cecal metabolomics sequencing and fecal 16s rRNA sequencing in *K. pneumoniae*-infected mice and controls and performed Spearman correlation analyses. We demonstrated that alterations in the composition of the gut microbiota led to profound changes in host metabolite abundances and that *Lactobacillus reuteri* and *Bifidobacterium pseudolongum* could be used as biomarkers for this bacterial pneumonia, which requires further study. Taken together, our findings provide a more refined picture of the relationship between the gut microbiota and remote organs or immune cells with respect to inflammatory response to extracellular invading pathogens.

The contributions made by the gut microbiota to the modulation of host immunity are largely attributed to microbial metabolism (57, 58), with bacteria being the largest of these contributors to the ecosystem (57). Bile acids, vitamins, and amino acids have emerged as significant and pleotropic signaling metabolites involved in the regulation of metabolism and inflammation through interactions with both microbiota and host receptors (15–17). Recent advances have identified SCFAs as an “indispensable linker” in host–microbiota communication networks. Initially described as a fuel resource for epithelial cells, hepatocytes, and peripheral tissues (59), they are now rapidly emerging as critical signals that directly influence immunity and cell function (25, 59, 60). Previous studies have described the beneficial effects of SCFAs in murine models of respiratory infection (61, 62). Here, we observed that the levels of three specific SCFAs, namely acetate, propionate, and butyric acid, were all markedly decreased in *K. pneumoniae*-infected mice compared to controls. Combining with microbiome analysis, we suspect that decreases in *Parabacteroides, Prevotella, Clostridium, Coprococcus*, and *Bifidobacterium* abundances may trigger decreases in SCFA levels and further trigger lung damage and several immune cascade reactions in the respiratory tract. The prime mechanisms involved in SCFA-immunomodulatory effects in macrophages involve inhibition of histone deacetylases (HDACs) (42, 63) and activation GPCRs (64, 65). Work conducted by Galvão et al. (28) suggested that the microbial metabolic sensor GPR43 modulated lung innate immunity against bacterial pneumonia through binding ligand acetate. Our work also showed that SCFAs (acetate, propionate, and butyrate) promoted host immune responses by enhancing the phagocytosis of macrophages, whereas the mechanisms still remained explored. Using BALB/c mice as well as less *K. pneumoniae*, Ciarlo et al. (66) showed that propionate does not alter susceptibility to infection *in vivo*. Consistent with our results, several proof-of-concept studies have demonstrated that levels of microbiome-derived metabolites, such as propionate, can influence lung immune and inflammatory responses *in vivo* (62, 67).

Our work supported earlier evidence that the antibacterial activity outside the intestinal lumen is programmed systemically by metabolites derived from the gut microbiota. It remains to be further investigated, however, whether there are other metabolites that contribute to these effects. In addition, how these microbial metabolites cooperate with the host internal antibacterial effectors to regulate the host immune system also remains to be elucidated. More importantly, it still remains to be determined whether the observed effects of gut microbiota depletion during infection with *K. pneumoniae* also apply to infections with other important causative agents of pneumonia. Characterizing microbial biomarkers has great potential for precision medicine and is a relatively simple way of translating microbiome research into clinical practice. Of clinical significance is how an altered gut microbiome and certain metabolites are associated with *K. pneumoniae*-induced pneumonia, and further studies will focus on whether it is possible to intervene in this bacterial pneumonia by targeting the gut microbiota.

## Data Availability Statement

The raw data supporting the conclusions of this article will be made available by the authors, without undue reservation, to any qualified researcher.

## Ethics Statement

The animal study was reviewed and approved by Anhui Medical University.

## Author Contributions

YLa and TW conceived and designed the study. TW, FX, CS, and HL performed the experiments and analyzed the data. YLa and TW wrote the manuscript. JL revised the manuscript. YLi and YG provided critical suggestions. JL and NL provided the funding. All authors reviewed the manuscript.

## Conflict of Interest

The authors declare that the research was conducted in the absence of any commercial or financial relationships that could be construed as a potential conflict of interest.
