# Supplementary material for: Alterations in the Gut Microbiome and Cecal Metabolome During Klebsiella pneumoniae-Induced Pneumosepsis
Source: Front Immunol. 2020 Jul 31;11:1331. doi: 10.3389/fimmu.2020.01331 (PMC7411141; doi:10.3389/fimmu.2020.01331)
Supplement: Supplementary file 1 [file Data_Sheet_1.PDF]

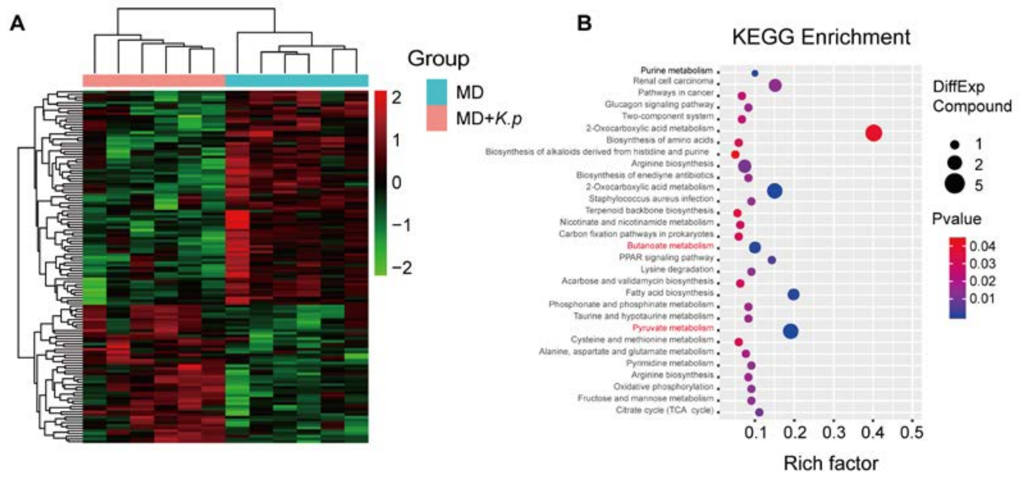

SUPPLEMENTARY FIGURE 1. Differential metabolites and KEGG pathway enrichment.

(**A**) Hierarchical clustering heatmap of metabolites identified in cecum contents from *K. pneumoniae*-infected, microbiota-depleted mice versus microbiota-depleted controls. Red denotes increased expression; green denotes decreased expression. **(B)** Selected examples of KEGG pathway enrichment. Disturbances on propionate and butyrate metabolisms are highlighted in red color. The *P* values are determined using Hypergeometric test and Benjamini-Hochberg FDR correction (**A**). MD, gut microbiota-depleted mice.

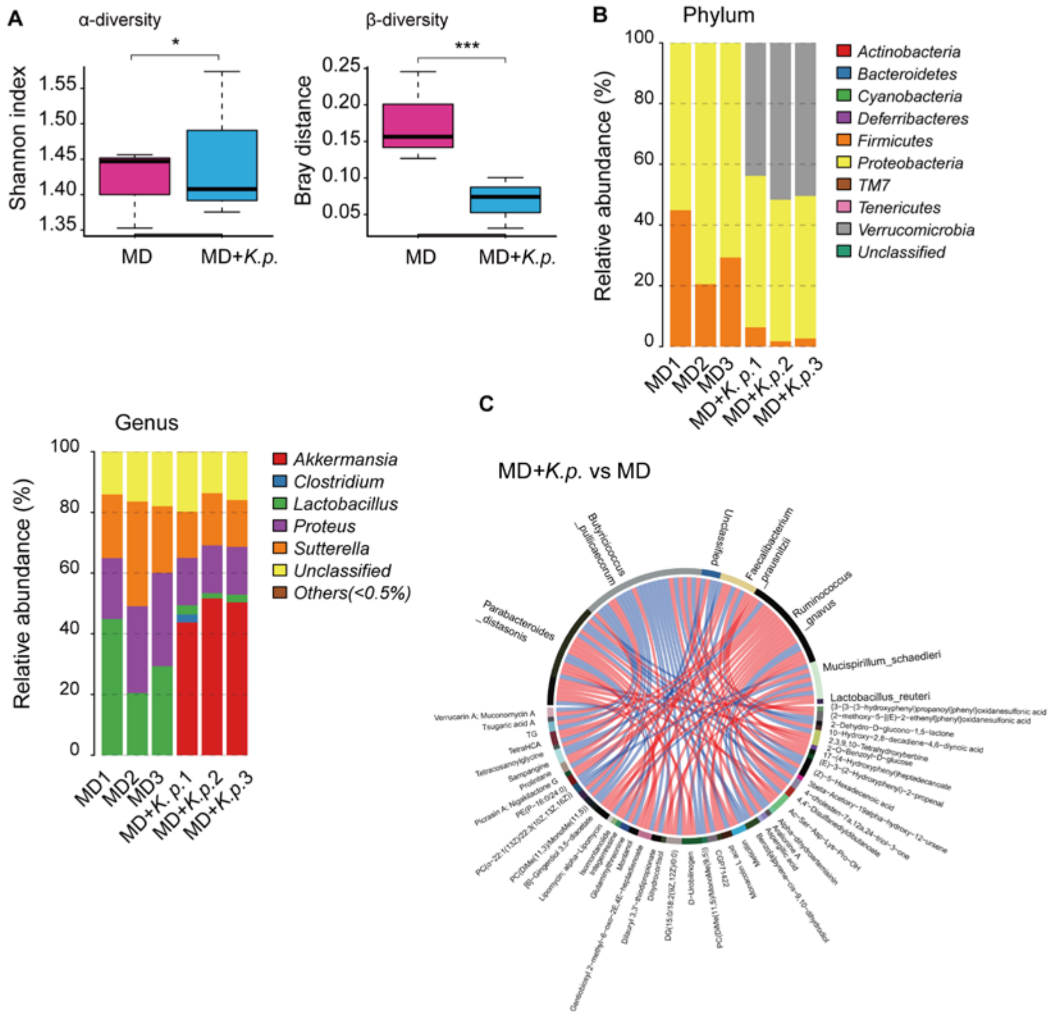

SUPPLEMENTARY FIGURE 2. Association of cecum metabolites and gut microbiomes.

(A) α-diversity (Shannon index) and β-diversity (Bray-Curtis similarity index) of 16S rRNA genes from *K. pneumoniae*-infected, gut microbiota-depleted mice and gut microbiota-depleted controls. **(B)** Discriminative OTUs abundance of taxonomic distributions at phylum and genus levels between *K. pneumoniae*-infected, gut microbiota depleted mice and gut microbiota-depleted controls. **(C)** Chord Chart of gut microbiota with corresponding metabolites at species level in *K. pneumoniae*-infected, gut microbiota-depleted mice and gut microbiota-depleted

controls. Conditions: r > 0.8 & $p < 0.05$. The *P* values are determined using two-tailed Wilcoxon rank-sum test (**B**). *$P < 0.05$, ***$P < 0.001$. MD, gut microbiota-depleted mice.
