# Supplementary material for: Alterations in the Gut Microbiome and Cecal Metabolome During Klebsiella pneumoniae-Induced Pneumosepsis
Source: Front Immunol. 2020 Jul 31;11:1331. doi: 10.3389/fimmu.2020.01331 (PMC7411141; doi:10.3389/fimmu.2020.01331)
Supplement: Supplementary file 2 [file Data_Sheet_2.PDF]

Supplementary Table S1. Regents and materials

Name	Comapny	Catalog
DMEM, high glucose	Gibico	Catalog# 11965-092
Opti-MEM™ I Reduced Serum Medium	Gibico	Catalog# 31985070
RPMI 1640 Medium	Gibico	Catalog# 31870082
Fetal Bovine Serum	Gibico	Catalog# 16000-044
Sodium Butyrate	Sigma Aldrich	Catalog# 303410-100G
Sodium Acetate	Sigma Aldrich	Catalog# S2889-250G
Sodium Propionate	Sigma Aldrich	Catalog# P1880-100G
Penicillin/streptomycin	Sigma Aldrich	Catalog# P4333-100ML
Gentamicin	Sigma Aldrich	Catalog# G1397-10ML
Ampicillin	Sigma Aldrich	Catalog# A9518-25G
Neomycin sulfate	Sigma Aldrich	Catalog# N6386-100G
Metronidazole	Sigma Aldrich	Catalog# M3761-25G
Puromycin Dihydrochloride	Invitrogen	Catalog# A1113802
CFSE	Invitrogen	Catalog# 65-0850-84
Lipofectamin2000	Invitrogen	Catalog# 11668027
Hoechst 33258	Sigma	Catalog# 861405
cOmplete™ Protease Inhibitor Cocktail	Roche	Catalog# 4693116001
TRIzol™ Reagent	Invitrogen	Catalog# 15596018
Lipopolysaccharide (LPS)	Sigma Aldrich	Catalog# L2630-10MG
